# Effectiveness and molecular interactions of the clinically active mTORC1 inhibitor everolimus in combination with tamoxifen or letrozole *in vitro *and *in vivo*

**DOI:** 10.1186/bcr3330

**Published:** 2012-10-17

**Authors:** Lesley-Ann Martin, Sunil Pancholi, Ian Farmer, Stephanie Guest, Ricardo Ribas, Marion T Weigel, Allan M Thornhill, Zara Ghazoui, Roger A'Hern, Dean B Evans, Heidi A Lane, Stephen R Johnston, Mitch Dowsett

**Affiliations:** 1 Breakthrough Breast Cancer Research Centre, Institute of Cancer Research, London, SW3 6JB UK; 2 McElwain Laboratory, Institute of Cancer Research, London, UK; 3 Clinical Trial Unit, Institute of Cancer Research, Surrey, SM2 5NG, UK; 4 Novartis Institutes for BioMedical Research, CH-4002, Basel, Switzerland; 5 The Royal Marsden Hospital, London, SW3 6JB, UK

## Abstract

**Introduction:**

Strategies to improve the efficacy of endocrine agents in breast cancer (BC) therapy and to delay the onset of resistance include concomitant targeting of the estrogen receptor alpha (ER) and the mammalian target of rapamycin complex 1 (mTORC1), which regulate cell-cycle progression and are supported by recent clinical results.

**Methods:**

BC cell lines expressing aromatase (AROM) and modeling endocrine-sensitive (MCF7-AROM1) and human epidermal growth factor receptor 2 (HER2)-dependent *de novo *resistant disease (BT474-AROM3) and long-term estrogen-deprived (LTED) MCF7 cells that had acquired resistance associated with HER2 overexpression were treated *in vitro *and as subcutaneous xenografts with everolimus (RAD001-mTORC1 inhibitor), in combination with tamoxifen or letrozole. End points included proliferation, cell-cycle arrest, cell signaling, and effects on ER-mediated transactivation.

**Results:**

Everolimus caused a concentration-dependent decrease in proliferation in all cell lines, which was associated with reductions in S6 phosphorylation. Everolimus plus letrozole or tamoxifen enhanced the antiproliferative effect and G_1_-accumulation compared with monotherapy, as well as increased phosphorylation (Ser^10^) and nuclear accumulation of p27 and pronounced dephosphorylation of Rb. Sensitivity was greatest to everolimus in the LTED cells but was reduced by added estrogen. Increased pAKT occurred in all circumstances with everolimus and, in the BT474 and LTED cells, was associated with increased pHER3. Decreased ER transactivation suggested that the effectiveness of everolimus might be partly related to interrupting cross-talk between growth-factor signaling and ER. In MCF7-AROM1 xenografts, letrozole plus everolimus showed a trend toward enhanced tumor regression, versus the single agents. In BT474-AROM3 xenografts, everolimus alone was equally effective at reducing tumor volume as were the combination therapies.

**Conclusions:**

The results provide mechanistic support for recent positive clinical data on the combination of everolimus and endocrine therapy, as well as data on potential routes of escape via enhanced HER2/3 signaling. This merits investigation for further improvements in treatment efficacy.

## Introduction

About 80% of primary breast cancer (BC) is estrogen-receptor alpha positive (ER^+^) and proliferates in response to estrogen (E). E mediates its effect by binding to ER, which in turn regulates transcription of target genes controlling proliferation and cell survival. Clinically, patients are treated with endocrine agents such as tamoxifen, which competes with E for the ER or aromatase inhibitors (AIs), which block the conversion of androgens to E. The most effective approach in postmenopausal patients is with AIs, but, as with other treatments, resistance to these agents develops in many cases. Studies in model systems indicate that this resistance may often depend on the acquisition of enhanced cross-talk between ER and growth-factor pathways that allows the disease to circumvent the need for steroid hormones [1].

In BC, the PI3K/AKT pathway modulates responses to signals, communicated through the ER and the HER family of receptors [2]. This pathway is important in the clinical sensitivity of BC to antiendocrine therapy [3-6]. *In vitro *studies have implicated AKT in the ligand-independent phosphorylation of the ER and subsequent resistance to tamoxifen [7,8]. Similarly, elevated levels of AKT have been shown to change the genome-wide binding pattern of ER, effectively altering the ER program [9]. These data suggest that signaling partners downstream of PI3K/AKT may provide potential therapeutic targets.

One rational possibility is mTOR, which exists in mammalian cells as two protein complexes; mTORC1 (containing raptor) and mTORC2 (containing rictor). mTORC1 regulates cell-cycle progression (the key effectors of endocrine therapy) by enhancing translation initiation and/or the stability of cell-cycle regulatory proteins, such as D-type cyclins [10], c-*myc *[11], p27^Kip1 ^[12], and p21^Waf1/Cip1 ^[13]. The two direct targets of mTORC1 are p70 S6 kinase and 4E-BP1, which mediate its effect on protein translation. Activation of mTORC1, in response to nutrient availability and activation of the PI3K/AKT pathway, results in the hyperphosphorylation of 4E-BP1 and the release of eIF4E, which, together with eIF4G, form a functional eIF4F mRNA cap binding complex and initiates translation. p70 S6 phosphorylates the 40S ribosomal subunit protein S6 and stimulates the translation of the 5' oligopyrimidine tract containing mRNAs [14]. Several of these cell-cycle regulators are dysregulated in BC, including eIF-4E [15], p27 [16], D-type cyclins [17], and c-*myc *[18]. Hence, mTORC1 may provide a novel target for the treatment of breast tumors that are endocrine resistant [19].

Evidence suggests that the mTORC1 inhibitor rapamycin, and its derivatives (rapalogs), may have some antitumorogenic activity [19,20]. Rapamycins/rapalogs are allosteric inhibitors that, when in complex with the immunophilin FKBP12, target the FRB domain adjacent to the catalytic site of mTORC1, leading to inactivation of p70 S6 kinase and activation of 4E-BP1 as a repressor of cap-dependent translation; resulting in the suppression of global protein synthesis [21]. Until recently, rapalogs showed modest clinical activity in BC [22,23].

Lately, however, two clinical studies reported substantially greater activity of the rapalog everolimus (RAD001) in the metastatic setting, when administered after prior treatment with an AI. First, in the TAMRAD study, median time to tumor progression (TTP) was 4.5 months (95% confidence interval (CI), 3.7 to 8.7) with tamoxifen and 8.5 months (95% CI, 6.01 to 13.9) with everolimus plus tamoxifen [24]. BOLERO-2 found that the median TTP with exemestane alone of 4.1 months after failure of nonsteroidal AI was extended to 10.6 months, a result so positive that it required early cessation of the trial [25].

We report here that in isogenic derivatives of MCF7 cells, the activity of everolimus is enhanced after acquisition of resistance to E-deprivation, together with mechanistic data that improve understanding of this enhanced activity. We also report xenograft studies of the combination of everolimus (RAD001) with the AI letrozole and parallel studies in the ER^+ ^BT474 cell line, whose endocrine resistance depends on HER2 amplification that is associated with response to rapalogs [26]. The results provide mechanistic support for recent positive clinical data on the combination of RAD001 and endocrine therapy, as well as data on potential routes of escape, via enhanced HER2/3 signaling, that merit investigation for further improvements in treatment efficacy.

## Methods

### Antibodies

These companies provided the following substances: Cell Signaling Technology, New England Biolabs, Hertforshire, UK (phospho-AKT^ser473^, AKT, ERK1/2, phospho-ERα^ser118^, phospho-ERα^ser167^, phospho-S6^ser240/244^, phospho-HER3^tyr1289^, phospho-p27^ser10^, p27, phospho-Rb^ser807/811^, Cyclin D3); Millipore (phospho-HER2^tyr1248^, HER2, HER3, IRS1, IRS2); Sigma, Poole, Dorset UK (phospho-ERK1/2^thr202/tyr204^, actin); Santa Cruz Biotechnology, Santa Cruz, USA (IGF1Rβ); Novacastra Laboratories, Newcastle upon Tyne, UK (ER). HRP-conjugated secondary antibodies were obtained from Amersham Pharmacia, Amersham UK. 17 β-Estradiol (E2) and 4-hydroxytamoxifen (4-OH tamoxifen) were obtained from Sigma Poole, Dorset, UK; RAD001 and letrozole were synthesized in the laboratories of Novartis Pharma AG, Basel, Switzerland. All chemicals, unless otherwise stated, were molecular grade and purchased from Sigma, Poole, Dorset UK. All tissue-culture-grade plastics were purchased from Thermo Fisher Scientific Nunc, Leicestershire UK.

### Tissue culture

MCF7-AROM1 and BT474-AROM3 were derived from parental cell lines (obtained from American Type Culture Collection) to stably express *CYP19 *(AROM) [27,28]. These modified cell lines were given the suffix AROM to distinguish them from the parental cells. AROM cells were maintained in phenol red-containing RPMI 1640 medium containing 2 m*M *glutamine, 10 μg/ml insulin, and 10% (vol/vol) fetal bovine serum (FBS) supplemented with 1 mg/ml G418. MCF7 cells that had adapted to long-term estrogen deprivation (LTED) were maintained in phenol red-free RPMI 1640 medium containing 2 m*M *glutamine, 10 μg/ml insulin supplemented with 10% (vol/vol) dextran-coated charcoal-stripped FBS (DCC-FBS) [29], referred to as DCC. For all experiments, cells lines were stripped of steroids for 3 days before seeding by culturing in DCC in the absence of insulin.

### Cell-proliferation assays

Cell lines were seeded into 12-well plates at densities between 1 and 4 × 10^4 ^cells per well. Cell monolayers were left to acclimatize for 24 hours before treatment with the drug combinations indicated for 6 days, with daily changes. Cell number was determined by using a Z1 Coulter Counter (Beckman Coulter, UK). The combination effects between RAD001 and 4-OH-tamoxifen or letrozole were analyzed by using isobolograms. To determine the nature of the interaction between RAD001 and letrozole or 4-OH-tamoxifen, combination studies were performed by using Chou and Talalay's constant ratio combination design and quantified by using Calcusyn software (BIOSOFT, Cambridge, UK) [30]. The combination indices (CI; mean and SD) for 50%, 75%, and 90% growth inhibition were obtained by using mutually nonexclusive Monte Carlo simulations, and statistical tests were applied (unpaired, two-tailed Student *t *test) to determine whether the CI values at multiple effect levels were significantly different from CI = 1. In this analysis, CI scores significantly lower than 1 were defined as synergistic; CI > 1, as antagonistic; and a CI = 1, as additive. Experiments were set up in triplicate.

### Transcription assay

Cell lines were seeded in 24-well plates at 7 × 10^4 ^cells per well in DCC medium for all cell lines except BT474, which was seeded at 1 × 10^5 ^cells per well. Twenty-four hours later, monolayers were transfected with Fugene (Roche, UK) with 0.1 μg of EREIItkluc and 0.1 μg of pCH110 overnight, before treatment with the drugs indicated. After 24 hours, luciferase (Promega, UK) and β-galactosidase (Galacto Star; PE Biosystems, UK) activities were measured by using a luminometer.

### Western blotting

Cell monolayers were extracted as described previously [31]. Protein concentrations were quantified by using BioRad protein assay kit (Bio Rad, UK). Proteins (50 μg) were resolved with SDS-PAGE and transferred to nitrocellulose filters (Schleicher and Schuell, UK). Filters were probed with specific antibodies as indicated. Immune complexes were detected by using the Ultra-Signal chemiluminescence kit from Pierce and Warriner (Chester, UK).

### Cell-cycle effects of RAD001 alone or in combination with endocrine agents

Cells were seeded into 10-cm dishes. Monolayers were treated with the drug combinations indicated for 24 hours. Cells were pulse-labeled with 10 μ*M *bromodeoxyuridine for 2 hours and then fixed and stained with anti-bromodeoxyuridine-conjugated FITC (Becton Dickinson) and propidium iodide. Fluorescence-activated cell signaling (FACS) was used to analyze changes in the cell cycle. To assess the effect on cell-cycle regulatory proteins, similarly treated cell monolayers were lysed and subjected to immunoblotting at the same time.

### Immunofluorescence

Cells were prepared as described previously [32]. After 24 hours of treatment with the drugs indicated, cells were fixed and incubated with a monoclonal anti-human p27 antibody (DakoCytomation), as previously described [32]. Cells were then incubated in the presence of Alexa Fluor 488-conjugated goat anti-mouse IgG secondary antibody (Molecular Probes), counterstained with TO-PRO-3 (Invitrogen), and mounted onto glass slides by using Vectashield mounting medium (Vector Laboratories). Images were collected sequentially in two channels on a Leica TCS SP2 confocal microscope (Milton Keynes, UK). The p27-positive nuclei were counted in each image by using the count tool in Adobe Photoshop CS6 Extended and were expressed as a percentage of total nuclei present (represented by DAPI-stained nuclei). Values shown are mean percentages ± standard deviation.

### Human tumor xenografts

Experiments were carried out in accordance with Home Office guidelines and approved by the Institute of Cancer Research Ethics Committee. Female Ncr Foxhead nude mice were kept under sterile conditions (eight per cage) with free access to food and water. Mice were ovariectomized and then allowed to acclimatize for 7 to 14 days. MCF7 AROM 1 and BT474 AROM 3 xenografts were initiated by inoculation of 100-μl cell suspension containing 10^7 ^cells in basement membrane matrix (Matrigel; BD Biosciences) into the right flank. Growth was maintained by androstenedione support through intradermal injection of androstenedione pellets (dose, 1.5 mg over a 60-day period; Innovative Research of America, Sarasota, FL, USA). Tumors were grown to approximately 7- to 8-mm diameter and assigned to treatment groups with no statistically significant difference in mean volume before treatment (Kruskal-Wallis ANOVA, MCF7 AROM 1, *P *= 0.14; and BT474-AROM3, *P *= 0.34). Mice were continued with androstenedione support and randomized to receive daily doses of vehicle (10% *N*-methyl-pyrollidone (NMP)/90% polyethylene glycol (PEG300), RAD001 (in 10% NMP/90% PEG300), tamoxifen (3.3 mg/ml in 10% NMP/90% PEG300), letrozole (0.17 mg/ml in 10% NMP/90% PEG300), or RAD001 in combination with tamoxifen or letrozole. All drugs were administered by oral gavage and were given daily for a total of 24 days.

Tumor growth was assessed twice weekly in all control and treatment arms by caliper measurements of the two largest diameters. Volumes were then calculated according to the formula: a × b^2 ^× π/6, where a and b are orthogonal tumor diameters. Tumor volumes were then expressed as fold change in volume at the start of treatment.

Overall statistical difference was calculated by using the Kruskal-Wallace test, and the statistical differences between individual treatment arms was calculated by using the Mann-Whitney test.

## Results

### Effect of RAD001 alone or in combination with endocrine therapy on cell growth

To enable the study of an AI in combination with everolimus, we used our MCF-7 and BT474 cells that had been genetically engineered to express aromatase (MCF7-AROM1 and BT474-AROM3) [27,28] and provided 10 n*M *androstenedione as growth support. Our long-term estrogen-deprived MCF7 cell line (LTED) was used to model acquired resistance to an AI. These cells show increased expression of HER2 but do not express aromatase (see Additional File 1) [31].

**Figure 1:**
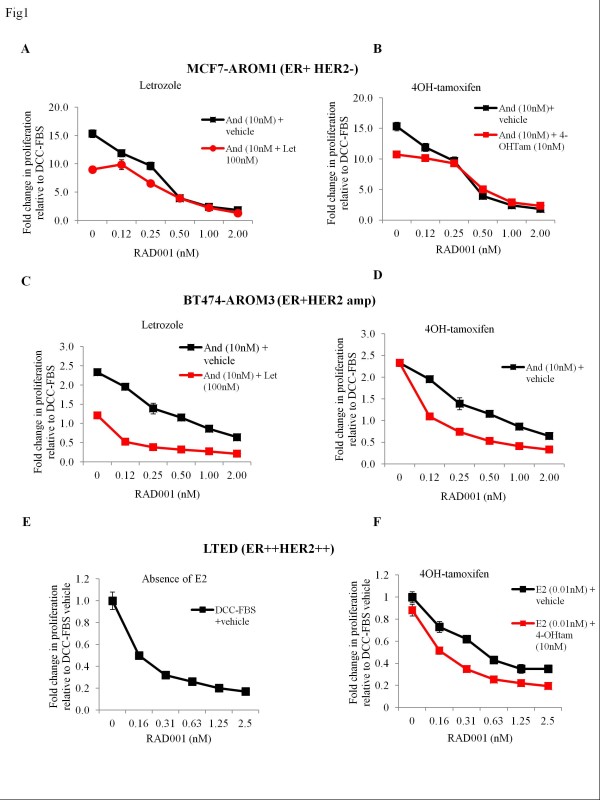
**Antiproliferative effects of RAD001, 4-OH tamoxifen, and letrozole in endocrine sensitive and resistant breast cancer cell lines**. Cells were treated with a standard concentration of androstenedione (10 n*M*) and doubling concentrations of RAD001 ± letrozole (100 n*M*) or 4OH-tamoxifen (10 n*M*). After 6 days of treatment, cell numbers were analyzed by using a Coulter counter. **(A, B) **MCF7-AROM1. **(C, D) **BT474-AROM3. **(E) **LTED cells treated with doubling concentrations of RAD001 in the absence of exogenous E2. **(F) **LTED cells treated with a standard concentration of E2 (0.01 n*M*) and doubling concentrations of RAD001 ± 4-OH tamoxifen (10 n*M*). Data are expressed as fold-change relative to DCC-FBS to show the increase in proliferation in response to androstenedione. Data shown are representative of three independent experiments. Error bars represent ± SEM.

RAD001 alone caused a concentration-dependent decrease in proliferation in all the cell lines tested (Figure 1A through 1F; black line on each graph). The median inhibitory concentration (IC_50_) for RAD001 was between 0.25 and 0.5 n*M *for MCF7-AROM1 in the presence of androstenedione and 0.5 n*M *for BT474-AROM3 (Figure 1A-D, black line) in the presence of androstenedione. The LTED cell line showed the greatest sensitivity, with an IC_50 _of 0.2 n*M *in the absence of exogenous E2 (Figure 1E, black line) versus 0.6 n*M *RAD001 in the presence of E2 (Figure 1F, black line).

The effect of doubling concentrations of RAD001 in combination with letrozole (100 n*M*) or 4-OH tamoxifen (10 n*M*) was assessed; the concentrations of each of the endocrine agents were close to their mean plasma levels obtained at the recommended doses of 2.5 mg/day letrozole or 20 mg/day tamoxifen. It should be noted that although 4-OH tamoxifen is a major active metabolite of tamoxifen, other metabolites may contribute to the clinical activity of this agent. Both letrozole and 4-OH tamoxifen alone decreased proliferation compared with androstenedione in MCF7 AROM1 cells, and a modest extra benefit was noted when added to RAD001 (Figure 1A and 1B). BT474-AROM3 cells showed sensitivity to letrozole alone but were resistant to 4-OH tamoxifen (Figure 1C and 1D). Of note, the combination of letrozole or 4-OH tamoxifen with doubling concentrations of RAD001 showed greater efficacy than RAD001 alone.

The LTED cells were used to model the cessation of AI at relapse by the addition of 0.01 n*M *E2. RAD001 was marginally more effective in the absence of added E2 (IC_50_, 0.2 n*M *(Figure 1E) versus IC_50 _0.63 n*M *in the presence of E2 (Figure 1F)). Similar to the BT474-AROM3 cells, addition of 4-OH tamoxifen improved the efficacy of RAD001 (Figure 1F).

**Figure 2:**
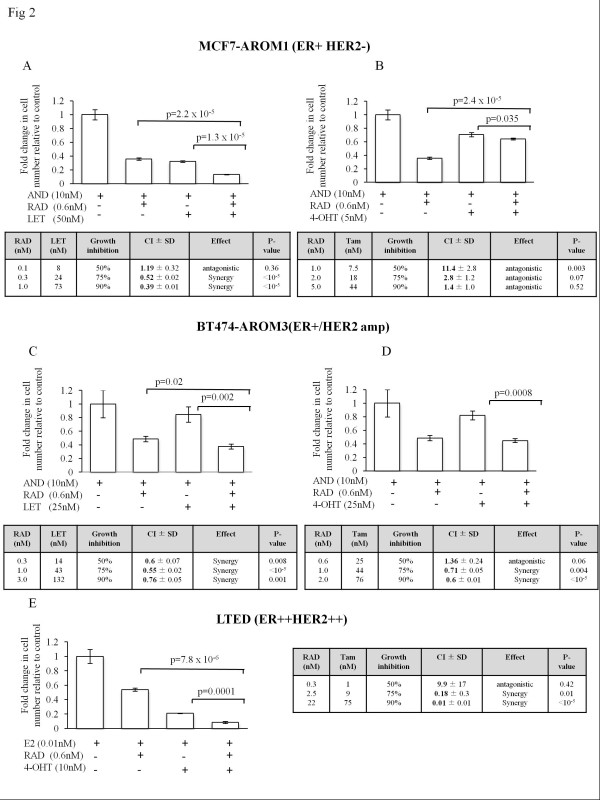
**Formal assessment of RAD001 combination index**. Cell lines were treated as described with IC_50 _concentrations of each drug alone or in combination, as indicated in the graphs. For Combination Index Tables shown, each graph MCF7-AROM1 **(A, B) **and BT474-AROM3 **(C, D) **were treated over a 6-day period in the presence of 10 n*M *androstendione with equipotent doses of letrozole, 4-OH tamoxifen, and RAD001. In the case of LTED cells **(E)**, similar analyses were carried out in the presence of E2 (0.01 n*M*), RAD001, and 4-OH tamoxifen at fixed ratios of the two agents. CIs were derived by using Calcusyn software, and significance was calculated as detailed in Methods.

We subsequently conducted formal assessment of the interaction between letrozole and 4-OH tamoxifen with RAD001. Calcusyn software was used to establish the IC_50 _dose of 4-OH tamoxifen, letrozole, and RAD001 for each of the cell lines. These were then combined in equipotent fixed-dose ratios. The antiproliferative effect of the drugs at their IC_50 _values alone and in combination is shown in Figure 2A through 2E. The tables are derived from equipotent doses of the drugs giving 50%, 75%, and 90% growth inhibition. Although from our initial analyses, enhancement of the antiproliferative effect of RAD001 was seen when combined with the endocrine agents in all circumstances (Figure 1), formal estimates showed a variety of interactions. In the MCF7-AROM1 cells, RAD001 was predominantly synergistic when used with letrozole, as indicated by combination indices (CIs) < 1 at 75% and 90% growth inhibition (Figure 2A). However, RAD001 was antagonistic with 4-OH tamoxifen at all doses tested CI > 1 (Figure 2B). In contrast, strong synergy was seen with 4-OH tamoxifen in the LTED cells with CIs < 1 at 75% and 90% growth inhibition (Figure 2E). The HER2-amplified BT474-AROM3 cells showed synergy with almost all doses of both letrozole (Figure 2C) and 4-OH tamoxifen (Figure 2D).

### RAD001 inhibits mTORC1 signaling but increases pAKT, pERK1/2, and pHER3

**Figure 3:**
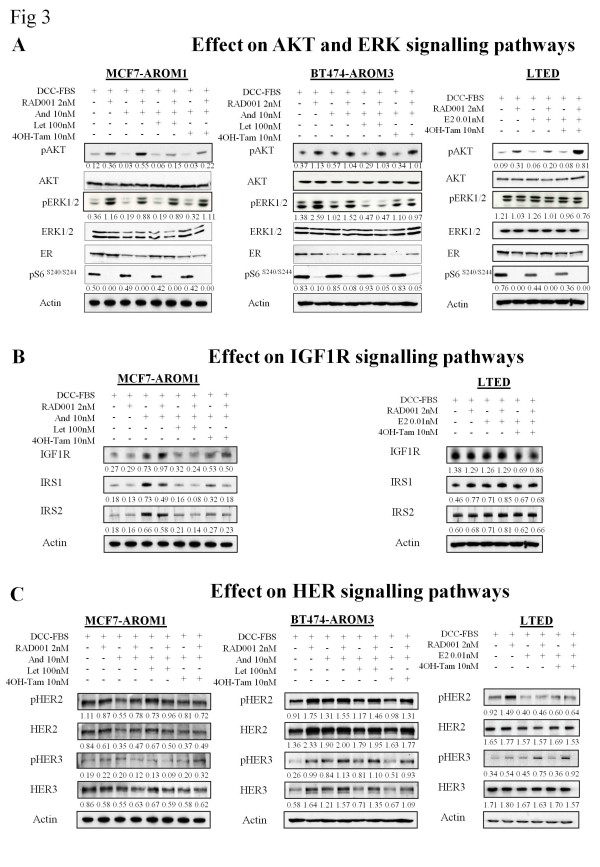
**RAD001 in combination with endocrine therapy induces increased pAKT, pERK1/2, and pHER3**. Steroid-depleted aromatase-expressing cells were treated for 24 hours with the drug combinations indicated. Whole-cell extracts were assessed for expression of various proteins indicated by using immunoblotting. **(A) **Effect of RAD001 alone or in combination with the endocrine agents on S6 kinase, ERK1/2, and AKT for the selected cell lines. **(B) **Effect of RAD001 on IGF1-R signalling. **(C) **Effect of RAD001 on HER signaling. Figures below each panel, where shown, represent semiquantitative changes in protein expression relative to actin.

To investigate the effect of RAD001 on cell signaling, MCF7-AROM1, BT474-AROM3, and LTED cells were treated for 24 hours with RAD001 ± letrozole or 4-OH tamoxifen (Figure 3). Phosphorylation of S6 at Ser^240/244 ^was dramatically suppressed by RAD001 alone or in combination with the endocrine agents in all cell lines (Figure 3A). In contrast, RAD001 alone or in combination increased the level of pAKT in each of the cell lines.

The combination of RAD001 and androstenedione ± 4-OH tamoxifen or letrozole increased pERK1/2 in MCF7-AROM1 cells (Figure 3A). Similarly, albeit to a far lesser extent, RAD001 increased pERK1/2 in both the DCC- and androstenedione-treated BT474-AROM3 cells. Letrozole treatment suppressed pERK1/2 similar to the MCF7-AROM1, but no increase in expression of pERK1/2 was seen with the addition of RAD001. Of note, altered expression of pERK1/2 was not evident in the LTED cells (Figure 3A). As increases in pAKT have been associated with alterations in IGF-1R signaling [33], we assessed the effect of RAD001 ± endocrine therapy on expression of IGF-1Rβ, IRS1, and IRS2 (Figure 3B). The MCF7-AROM1 cell line showed increased levels of IGF-1Rβ, IRS1, and IRS2 in response to androstenedione, which were suppressed by letrozole and 4-OH tamoxifen. Addition of RAD001 suppressed further the levels of IRS1, an observation in contrast to that previously reported [33]. At present, this observation remains unexplained. IRS2 remained unchanged in response to RAD001 in the MCF7-AROM1. Addition of RAD001 to LTED cells caused a slight, but expected, increase in IRS1 and not IRS2 (Figure 3B). IGF-1R expression in the BT474-AROM3 cells was extremely low, and neither IRS1 nor IRS2 was detectable with Western blot (data not shown). Assessment of the impact of RAD001 on HER signaling (Figure 3C) showed that RAD001 ± endocrine therapy increased pHER2, pHER3, total HER2, and HER3 expression in the BT474-AROM3. The LTED cells showed a marked increase in pHER2 and total HER2 in response to RAD001 in the absence of E2. In keeping with the BT474-AROM3, the LTED cells also showed a marked increase in pHER3 in response to RAD001, although no corresponding increase in total HER3 protein expression was evident. The MCF7-AROM1 cells showed no significant changes in either HER2 or HER3 under the conditions tested.

### RAD001 in combination with 4-OH tamoxifen or letrozole enhances G_1 _arrest and increases p27 phosphorylation and nuclear localization

**Figure 4:**
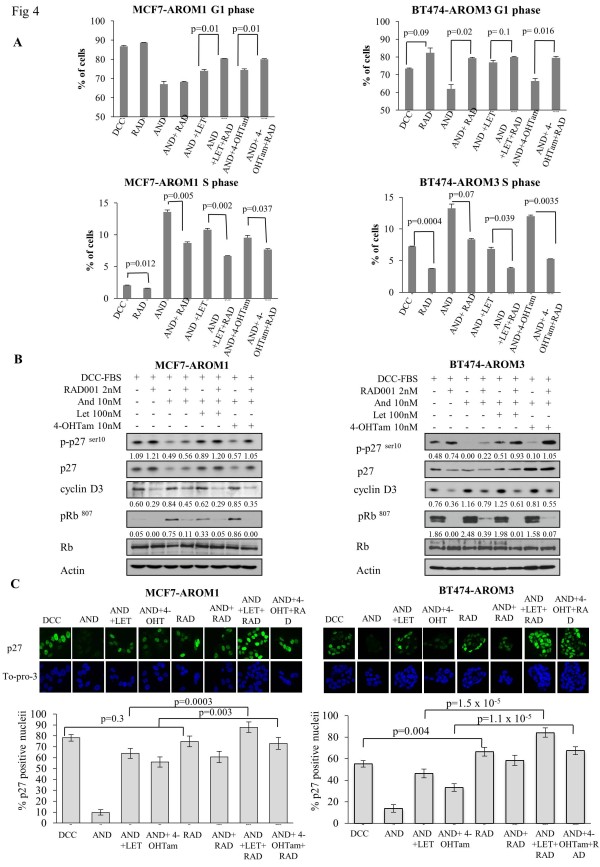
**RAD001 in combination with 4-OH tamoxifen or letrozole enhances G_1 _arrest compared with that with monotherapy**. **(A) **Steroid-depleted MCF7-AROM1 and BT474-AROM3 cells were treated for 24 hours with vehicle, androstenedione (10 n*M*), 4-OH tamoxifen (10 n*M*), or letrozole (100 n*M*) alone or in combination with RAD001 (2 n*M*). Cell cycle was monitored with FACS analysis of cells stained with BrdU and PI. Duplicate plates treated with the drug combination were harvested after 24 hours of treatment. **(B) **Whole-cell extracts were probed for phosphorylated p27^Kip1^, cyclin D3, and phosphorylated Rb. Figures below each panel, where shown, represent semiquantitative changes in protein expression relative to actin. **(C) **The effect of drug treatment on p27^Kip1 ^cellular localization was monitored with confocal microscopy. p27^Kip1 ^was visualized with Alexa 488-conjugated antibodies (green), whereas nuclei were counterstained with TO-PRO-3 (blue). Graphs represent percentage of total nuclei present (represented by DAPI-stained nuclei). Values shown are mean percentages ± standard deviation.

As mTORC1 is strongly implicated in the regulation of D-type cyclins [16] and p27 [12], the effect of RAD001 ± endocrine therapy on cell-cycle progression was assessed. Changes in the percentage of cells in G_2_/M were only modest (data not shown); therefore, we focused our analysis on S-phase and G_1_-phase alterations (Figure 4A). Androstenedione increased the percentage of cells in S-phase compared with control in both MCF7-AROM1 and BT474-AROM3. RAD001 in combination with letrozole or 4-OH tamoxifen increased the number of cells in G_1 _versus the monotherapies in both the MCF7-AROM1 (letrozole, 74%, versus letrozole + RAD001, 80%; *P *= 0.01; 4-OH tamoxifen, 75% versus 4-OH tamoxifen + RAD001, 80%; *P *= 0.01) and the BT474-AROM3 (letrozole, 77%, versus letrozole + RAD001, 80%; *P *= 0.1; 4-OH tamoxifen, 66%, versus 4-OH tamoxifen + RAD001, 80%; *P *= 0.016). Reciprocal changes were noted for the treatment effects on S-phase.

In the presence of androstenedione, increased p27^ser10 ^phosphorylation was evident in response to RAD001 and letrozole, as compared with androstenedione alone in both BT474-AROM3 and MCF7-AROM1. The combination of RAD001 ± either endocrine agent caused a marked increase in p27^ser10 ^phosphorylation in BT474-AROM3. Similarly, but to a lesser extent, p27^ser10 ^phosphorylation was also increased in MCF7-AROM1 in response to the combinations. A corresponding decrease in expression of cyclin D3 and pRb^807 ^in response to RAD001 ± endocrine therapy was also seen, with Rb phosphorylation in particular being more profoundly affected by combination treatment in both MCF7-AROM1 and BT474-AROM3 (Figure 4B).

AKT can phosphorylate p27 on threonine 157 (p27^kip1Thr-157^), suppressing nuclear import and subsequent p27-driven G_1 _arrest [34]; hence, confocal microscopy was used to detect nuclear p27. The combination of RAD001 ± letrozole or 4-OH tamoxifen significantly increased the number of nuclei positive for p27 compared with monotherapy in both cell lines (Figure 4C).

### The effect of RAD001 alone or in combination with endocrine therapy on ER-transactivation

**Figure 5:**
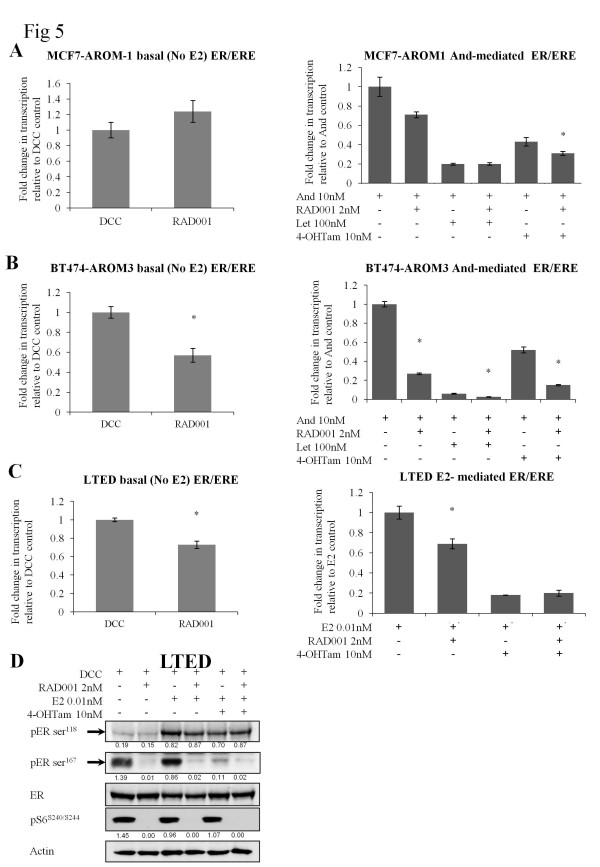
**Effects of RAD001 on ER-mediated transcription**. **(A, B) **Cell lines, co-transfected with EREIItkLuc and pCH110, were treated with RAD001 (2 n*M*) alone (basal ER-mediated transcription) or, to represent E2-mediated transcription, with a standard 10 n*M *concentration of androstenedione ± 4-OH tamoxifen (10 n*M*) or letrozole (100 n*M*) ± RAD001 (2 n*M*). **(C) **LTED cells were treated similarly, except that E2 (0.01 n*M*) was used in place of androstenedione. Luciferase activity was normalized by β-galactosidase from co-transfected pCH110. Normalized luciferase activity from triplicate wells was expressed relative to the vehicle-treated control. Bars represent ± SEM. *P < 0.05, derived from the comparison of vehicle (DCC) versus RAD001 for basal transcription or the endocrine agent alone versus the combination with RAD001 with Student unpaired *t *test. Effects were confirmed in two independent experiments. **(D) **LTED cells were treated as shown for 24 hours. Western blot was used to assess changes in phosphorylation of the ER in response to RAD001. Figures below each panel, where shown, represent semiquantitative changes in protein expression relative to actin.

MCF7-AROM1, BT474-AROM3, and LTED cells were transiently transfected with an ERE-luciferase reporter construct and treated with 4-OH tamoxifen or letrozole ± RAD001 (Figure 5) to assess whether the interactions between the drugs were related to effects on E-dependent transactivation. RAD001 had no significant effect on ER-mediated transactivation in the MCF7-AROM1 cells ± androstenedione or letrozole compared with the single agents (Figure 5A). However, 4-OH tamoxifen plus RAD001 reduced ER-mediated transcription by a further 30% compared with 4-OH tamoxifen alone. In contrast, in BT474-AROM3 and LTED cells, RAD001 caused a significant decrease in ER-mediated transcription in both the presence and the absence of an estrogenic signal (Figure 5B, C). Notably, the combination of RAD001 with both letrozole and/or 4-OH-tamoxifen further suppressed ER-mediated transactivation compared with the single agents in the BT474-AROM3 cells.

S6 kinase has been previously associated with the ligand-independent activation of the ER [35]; we therefore assessed the effect of RAD001 on the phosphorylation of ER in the LTED cells modeling acquired resistance. RAD001 alone and in combination with E2 ± 4-OH tamoxifen significantly reduced pERser^167 ^but had no impact on pERser^118 ^(Figure 5D).

### The effect of RAD001 in MCF7-AROM1 and BT474-AROM3 xenograft models

**Figure 6:**
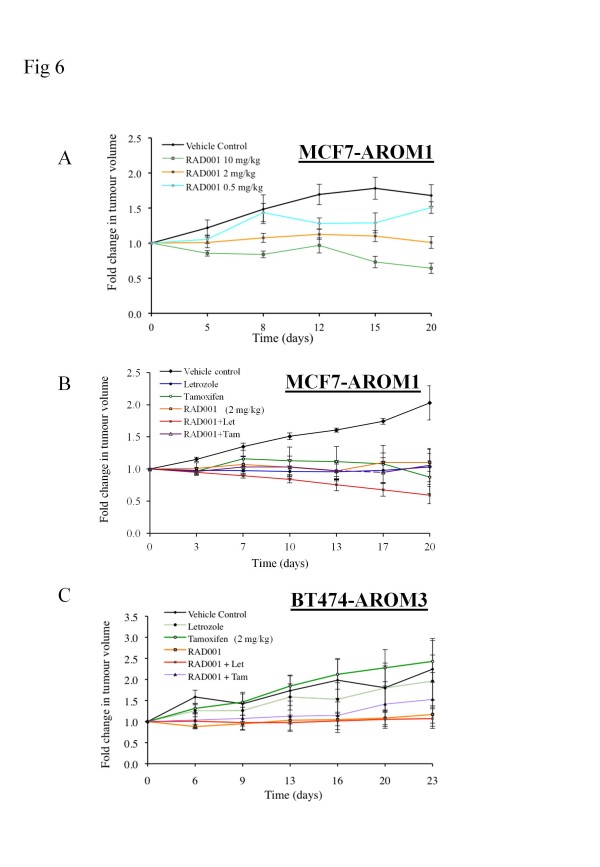
**Effect of RAD001, letrozole, and tamoxifen, alone or combination, on the growth of MCF7-AROM1 and BT474-AROM3 xenografts**. MCF7-AROM1 xenograft was grown in the presence of androstenedione. Once tumors reached 7 to 8 mm in diameter, animals were randomized to receive vehicle, 0.5 mg/kg (*n *= 6), 2 mg/kg (*n *= 6), or 10 mg/kg RAD001 (*n *= 6). Bars represent ± SEM. **(B) **MCF7-AROM1 xenografts were grown as described earlier. Once tumors reached 7 to 8 mm in diameter, animals were randomized to receive vehicle (*n *= 4), tamoxifen (*n *= 4), letrozole (*n *= 5), RAD001 (2 mg/kg) (*n *= 5), or a combination of agents (*n *= 5). **(C) **BT474-AROM3 grafts were grown as described earlier. Once tumors reached 7 to 8 mm in diameter, animals were randomized to receive vehicle (*n *= 8), tamoxifen (*n *= 7), letrozole (*n *= 8), RAD001 (2 mg/kg) (*n *= 8), or a combination of agents (*n *= 8). Tumors were measured twice weekly, and points represent mean change in tumor volumes over that of day 0. Bars represent ± SEM.

MCF7-AROM1 cells were injected subcutaneously into immunocompromised mice and maintained under androstenedione support. With this model, the effects of increasing doses of RAD001 on tumor growth versus the vehicle-treated control were studied. The mean fold-change in tumor volume for each treatment is shown in Figure 6A. Tumor volumes in the vehicle-treated mice increased over the study period (mean, 1.68 ± 0.15-fold on day 20). The mean daily growth rate, expressed as daily volume change relative to the vehicle group over the study period, was significantly reduced at concentrations of 2 mg/kg and 10 mg/kg RAD001 compared with the vehicle (*P *< 0.05). Based on these data, a concentration of 2 mg/kg RAD001, which appeared to provide stable disease, was selected for the combination studies with letrozole and tamoxifen (Figure 6B). Letrozole induced tumor stabilization (1.03 ± 0.27 fold-change at day 20 versus control, 2.03 ± 0.27). Similarly, both tamoxifen (0.87 ± 0.25) and RAD001 (1.10 ± 0.14) reduced tumor volume compared with the vehicle-treated control. Importantly, the combination of RAD001 with letrozole caused tumor regression (fold-change in tumor volume, 0.59 ± 0.14), whereas the combination with tamoxifen provided no clear benefit over the single agents (1.06 ± 0.26). However, the growth rate over the study period was not significantly different between the RAD001 and letrozole (*P *= 0.15) or tamoxifen (*P *= 0.971) groups. Although the growth rate in the mice treated with the combination of RAD001 and letrozole was significantly less than that with the vehicle (*P *= 0.016), no statistical difference was found between the combination and letrozole alone (*P *= 0.69).

The effects of RAD001 in combination with the endocrine agents were also investigated in a second xenograft model using BT474-AROM3 (ER^+^, HER2 amp) cells (Figure 6C). The mean tumor-volume fold change was 2.07 ± 0.7 at day 23 for the vehicle. However, in contrast to the previous model, neither letrozole nor tamoxifen reduced tumor volume, and, although not statistically significant, a trend was noted toward tamoxifen promoting tumor growth compared with the vehicle-treated control arm (fold-change, 2.42 ± 0.33, versus vehicle, 2.07 ± 0.7). Of note, RAD001 alone induced tumor stabilization (1.25 ± 0.33 fold-change at day 23 versus control, 2.07 ± 0.7). Assessment of the combination arms revealed no significant difference compared with RAD001 as a single agent (RAD001 versus letrozole + RAD001, *P *= 0.77; RAD001 versus tamoxifen + RAD001, *P *= 0.89). Furthermore, the combination of RAD001 and tamoxifen appeared to have less effect than did RAD001 alone, although this did not approach statistical significance

No significant alterations in body weight were found between the vehicle and any of the treatment arms (data not shown). LTED cells were unable to be established as xenografts, so data are not available.

## Discussion

RAD001 resulted in concentration-dependent decrease in proliferation in all cell lines tested, most markedly in the LTED. In combination with endocrine therapy, RAD001 enhanced the antiproliferative effect and G_1_-accumulation compared with monotherapy. This was associated with pronounced dephosphorylation of Rb and increased phosphorylation and nuclear accumulation of p27. RAD001 increased pAKT in all circumstances, which was associated with increased pHER3. Furthermore, RAD001 decreased ER transactivation, suggesting that the efficacy of RAD001 may relate to interrupting cross-talk between growth-factor signaling and ER, leading to decreased ER phosphorylation.

Over recent years, a drive has occurred toward the use of targeted agents for BC treatment. Both *in vitro *and *in vivo *models of endocrine-resistant BC suggest a shift from the dependence of tumor cells on the steroid-receptor pathways driving proliferation to dependence on growth-factor pathways. This allows the resistant tumor to circumvent the need for steroid hormone through downregulation of genomic ER function or by hypersensitivity to low levels of estradiol [36]. The PI3K pathway is strongly implicated in endocrine resistance, and agents that target kinases within this network have received significant interest [37]. A drive has been noted toward the rational combination of agents that target *de novo *resistance or seek to block acquired resistance. The combination of RAD001 with exemestane was recently found, in the BOLERO-2 trial [25], to be more effective than exemestane alone for the treatment of advanced BC after initial treatment with a nonsteroidal AI, but few data from laboratory models provide a mechanistic explanation.

A large body of evidence links the ER and AKT/mTORC1 pathways. Studies with CCI-779 show inhibitory effects on BC cell lines that either are E2-dependent, overexpress HER2, or lack expression of PTEN [38]. Further studies showed a good correlation between sensitivity to CCI-779 and AKT expression [39]. More recently, it was demonstrated that RAD001 in combination with letrozole was more effective at inhibiting the androstenedione-driven proliferation of both MCF7 and T47D breast tumor cells than was either drug alone [40].

Based on these findings, we aimed to assess the efficacy of RAD001 ± letrozole or 4-OH tamoxifen *in vitro *and *in vivo *in BC cell lines modeling endocrine-sensitive, acquired, and *de novo*-resistant disease that is dependent on HER2 overexpression. RAD001 inhibited the proliferation of all cell lines tested in a dose-dependent manner and increased the sensitivity of both BT474-AROM3 and LTED BC cells to E-deprivation. In the latter case, the data are analogous to those from the enhanced activity of RAD001 plus exemestane versus exemestane alone in BOLERO-2 [25]. Notably, our data in LTED cells indicate that maintained suppression of estrogens is likely to be important for the greatest benefit from RAD001. The LTED cells show markedly increased HER2 expression compared with MCF7 cells [31] and, along with the HER2-amplified BT474 cells, suggest that endocrine resistance due to HER2 overexpression may represent a particularly sensitive phenotype for targeting mTOR. Our data also imply that tamoxifen plus RAD001 may be an effective combination in tumors with acquired resistance to E-deprivation.

The function of ER as a transcription factor is modulated through phosphorylation; we therefore sought to determine the effect of RAD001 on ER-mediated transcription. Recent reports have shown that mTORC/S6K1 and ERK1/2/p90RSK contribute nonoverlapping inputs into ERα activation through Ser^167 ^phosphorylation [35]. This may account for the reported additive/synergistic effects of rapamycin and tamoxifen on MCF7 cell survival *in vitro *[41,42] and the observation that in tamoxifen-resistant cell lines, co-treatment with rapamycin *in vitro *or CCI-779 *in vivo *inhibited mTOR activity and restored tamoxifen sensitivity [43]. RAD001 significantly inhibited E2-mediated ER-transactivation in the HER-2 expressing cell lines, BT474-AROM3 and LTED, but not MCF7-AROM1. This would suggest that ER function may be dependent on cross-talk between HER2/mTORC/S6K1 and ER in the endocrine-resistant cell lines. In support of this, inhibition of S6K by RAD001 significantly reduced phosphorylation of ER Ser^167^, in keeping with previous studies [35,44] and resulted in a modest inhibitory effect on E2-driven phosphorylation of ER Ser^118^.

In MCF7-AROM1 xenografts, the combination of RAD001 and letrozole induced tumor regression, as opposed to the stabilization observed with the monotherapies, although analysis of growth rates did not show this to be statistically significant. Moreover, combination with 4OH-tamoxifen provided no clear benefit over the single agents. These data are consistent with the *in vitro *data with MCF7-AROM1 cells, where more profound combination effects were observed with RAD001 in combination with letrozole, supporting a combination of AI rather than tamoxifen with RAD001 for the first-line treatment of ER^+ ^BC.

In the BT474-AROM3 xenograft, RAD001 alone was superior to both letrozole and tamoxifen when provided as monotherapies, and no additional benefit was seen by treatment with RAD001 in combination with letrozole or tamoxifen. The lack of a combination effect may reflect the complexity of the tumor/stroma environment, which cannot be recapitulated *in vitro*, or may indicate that alternative doses and administration schedules should be evaluated. However, based on RAD001-associated increases in AKT phosphorylation *in vitro*, which was particularly evident with the combination treatments in this model, compensatory survival signals may be responsible for restricting the efficacy of the combination treatments *in vivo*. Inhibition of mTORC1 is known to induce upstream receptor tyrosine kinase signaling and to activate AKT [33]. Furthermore, inhibition of mTORC1 also leads to activation of the ERK signaling pathway [45]. This may have clinical implications, as some tumors from patients treated with RAD001 showed an increase in phospho-AKT and/or phospho-ERK, a phenomenon postulated to explain the comparatively modest clinical activity of rapamycins as single agents [33,45,46]. The absence of regression of the BT474 tumors in response to any of the treatments, although of mechanistic interest, has limited clinical significance, given that HER2-amplified tumors are now treated with HER2-targeted therapy, such as trastuzumab.

Further to address the potential escape routes from RAD001, we characterized the effect of the treatments on the major signaling pathways in the cell-line models. The mTOR-activated kinase S6K1 is known to phosphorylate and destablize IRS1 and IRS2 in insulin-like growth factor (IGF)-responsive cells [33]. mTOR inhibition reduces S6K1 activity, causing an increase in IRS1/2 and enhanced activation of IGFR1-dependent Akt activity [33]. Of note, the IGF pathway regulates ER function via S6K [44], providing a strong link between mTOR and ER activity. The LTED cells showed a slight, but expected, increase in IRS1 in response to RAD001. However, both the LTED and BT474-AROM3 showed increased pHER3, which also correlated with enhanced pAKT. Previous studies have shown that the HER3/PI3K signaling pathway increases expression of survivin, an inhibitor of apoptosis in HER2-expressing cell lines, and is associated with resistance to laptinib and chemotherapy [47,48]. Although RAD001 has a substantial impact on the HER2-overexpressing cell lines, the enhanced HER3 signaling may impede its long-term efficacy. The activation of pAKT is recognized as a likely escape route from inhibition of mTORC1, and the data from this study indicate that this persists in combination with endocrine therapy. Dual targeting of mTOR and upstream HER pathways, along with endocrine therapy, is likely to be more effective.

## Conclusions

RAD001 in combination with endocrine therapy provides little further benefit compared with endocrine therapy alone in a model of hormone-sensitive ER^+ ^BC. In contrast, RAD001 was effective as monotherapy in ER^+ ^endocrine-resistant cells based on HER2 overexpression or amplification, and in those cells with acquired resistance, maintained E-deprivation was important for maximal effectiveness of RAD001. The benefit may reflect interruption of growth factor-dependent transactivation of ER. The results provide mechanistic support for recent positive clinical data on the combination of RAD001 and endocrine therapy, as well as data on potential routes of escape through enhanced HER2/3 signaling, which merit investigation for further improvements in treatment efficacy.

## Abbreviations

4-OH tam or 4-OHT: 4-OH tamoxifen; AI: aromatase inhibitor; AROM: aromatase; E2: estradiol; ER: estrogen receptor; ERE: estrogen response element; eIF4E: eukaryotic initial factor 4E; HER2: human epidermal growth factor receptor 2; IGF-1R: insulin-like growth factor receptor; IRS1 (IRS2): insulin substrate 1 and 2; LTED: long-term estrogen deprived; mTOR: mammalian target of rapamycin; wt: wild-type.

## Competing interests

R A, MTW, ZG, IF, SG, RR, AT, and SP have no conflict of interest that could be perceived as prejudicing the impartiality of the research reported. DBE is an employee of Novartis; HL is an employee of Novartis; SRJ receives research funding, honoraria for advisory boards and lecture fees from AstraZeneca, Novartis, and Pfizer; MD receives research funding, honoraria for advisory boards, and lecture fees from AstraZeneca, Novartis, and Roche; LAM receives academic research funding from AstraZeneca and Pfizer.

## Authors' contributions

LAM and MD conceived of the study, participated in its design and coordination, and drafted the manuscript. SP, IF, and MW carried out proliferation and protein analyses. RR and SG carried out FACs analysis and interpretation of cell-cycle data. ZG and RH performed the statistical analyses. AT performed xenograft experiments. SRJ, HAL, and DBE participated in the design of the study and provided critical revision of the manuscript. All authors read and approved the final manuscript.

## Supplementary Material

Additional File 1 **Comparative expression of HER2 in LTED versus BT474 cells**. Whole-cell extracts from MCF7, LTED, and BT474 cells were resolved with SDS-PAGE and immunoblotted for expression of HER2 and actin. Click here for file
