# Supplementary figures and images for: Effectiveness and molecular interactions of the clinically active mTORC1 inhibitor everolimus in combination with tamoxifen or letrozole in vitro and in vivo
Source: Breast Cancer Res. 2012 Oct 17;14(5):R132. doi: 10.1186/bcr3330 (PMC4053110; doi:10.1186/bcr3330)

## Slide 1
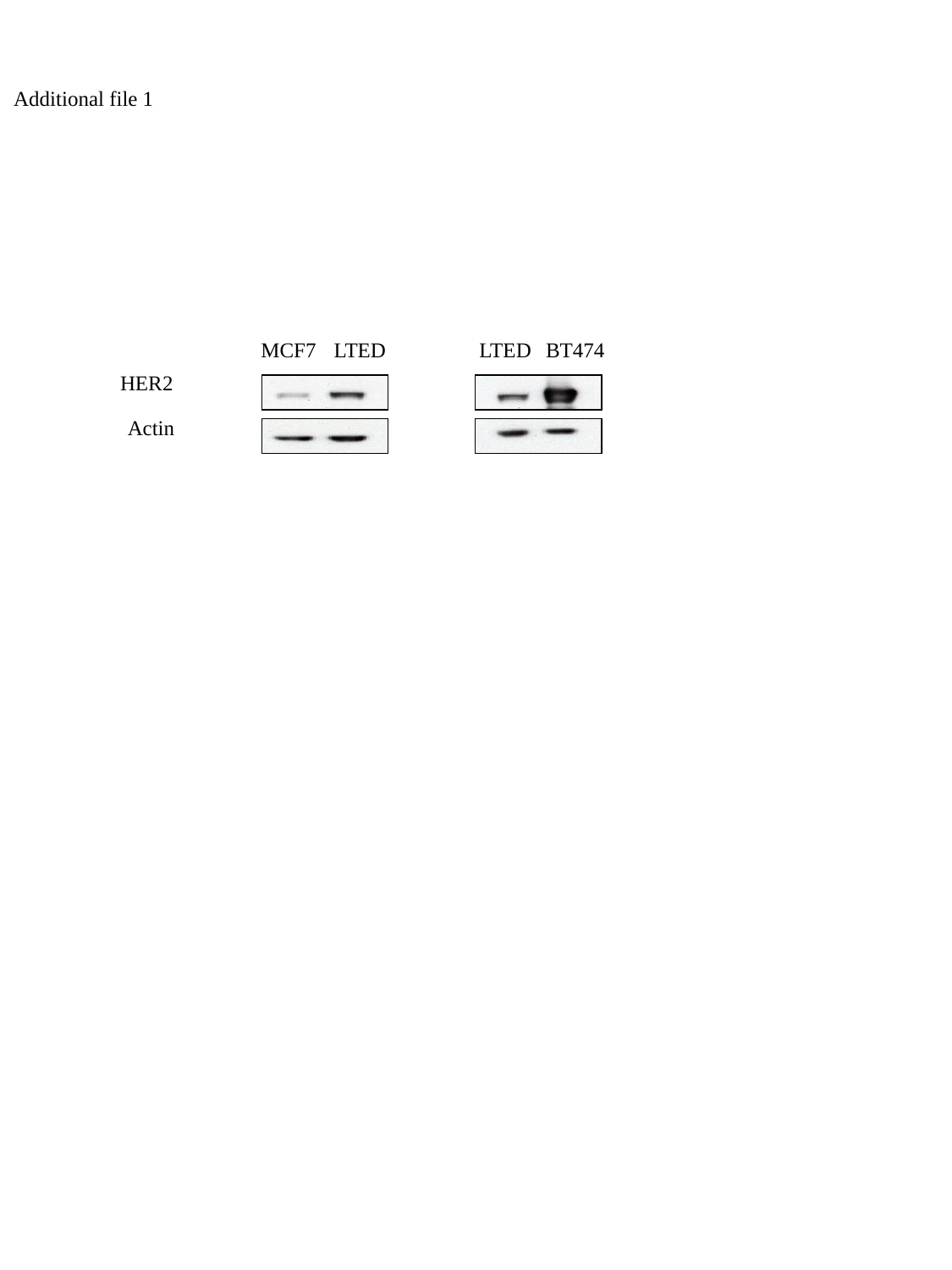

Additional file 1
MCF7
LTED
LTED
BT474
HER2
Actin

Supplement: Additional File 1 — Comparative expression of HER2 in LTED versus BT474 cells. Whole-cell extracts from MCF7, LTED, and BT474 cells were resolved with SDS-PAGE and immunoblotted for expression of HER2 and actin. [file bcr3330-S1.PPT]
